# Coatings Based on Phosphate Cements for Fire Protection of Steel Structures

**DOI:** 10.3390/ma14206213

**Published:** 2021-10-19

**Authors:** Cristina Andreea Vijan, Alina Badanoiu, Georgeta Voicu, Adrian Ionut Nicoara

**Affiliations:** Department of Science and Engineering of Oxide Materials and Nanomaterials, Faculty of Applied Chemistry and Materials Science, University Politehnica of Bucharest, 1-7 Gheorghe Polizu Street, 011061 Bucharest, Romania; cris_deea_vajan@yahoo.com (C.A.V.); georgeta.voicu@upb.ro (G.V.); adrian.nicoara@upb.ro (A.I.N.)

**Keywords:** fire protection, coating, phosphate cement, dead burned magnesite, partially calcined dolomite, steel

## Abstract

Fire events in buildings can cause losses to human life and important material damage, therefore a great deal of attention is paid nowadays to fire prevention. Buildings based on steel structures are especially affected in the event of a fire, due to the important loss of load-bearing capability when steel is heated at temperatures higher than 500 °C. Therefore, one possible method to mitigate the deleterious effect of fire is to protect steel structures from direct heating by applying protective coatings. In this paper, the ability of magnesium phosphate cement (MPC), based on dead burned magnesite and calcium magnesium phosphate cement (CMPC) coatings, to protect a steel substrate was assessed. CMPCs were obtained by mixing partially calcined dolomite with a KH_2_PO_4_ (MKP) solution, and in some cases, with a setting retarder (borax). The main mineralogical compounds assessed by X-ray diffraction and electronic microscopy (SEM-EDS) in CMPC are MgO, CaCO_3_, and K-struvite (KMgPO_4_·6H_2_O). The coatings based on MPC and CMPC, applied to steel plates, were tested in direct contact with a flame; the coatings of MPC and CMPC without the borax addition prevented the temperature increase of a metal substrate above 500 °C. No exfoliation of coatings (MPC and CMPC without borax addition) was noticed during the entire period of the test (45 min).

## 1. Introduction

Fire events in buildings can cause losses to human life and important material damages. Therefore, a great deal of attention is paid to the fire safety of buildings during the design and execution of constructions. Fire prevention methods include the use of active and passive fire protections along with increased fire prevention awareness [1]. Passive fire protection is designed to limit fires when they occur in buildings, and can consist of, among other methods, the use of fire-resisting construction materials or the modification of the usual construction materials in order to improve their fire resistance. This type of protection is particularly important for buildings based on metal-framed structures, due to the loss of steel’s load-bearing capability when heated at temperatures higher than 500 °C [1,2]. Therefore, this type of structure should be protected from direct heating in the event of a fire, by applying protective coatings. The use of coatings based on Portland cement (mortars or concretes) to protect the steel columns and beams is an attractive option due to the ease of application and low cost of the material; nevertheless, the use of these types of coatings also has serious limitations, one of the most important being their tendency to crack and dislocate at higher temperatures [1].

Among other types of inorganic binders used for the preparation of fire-protective coatings or composites is magnesium phosphate cement (MPC). This type of cement is obtained by mixing dead burnt magnesia with phosphoric acid or phosphate salts. Compared with Portland cement, MPC has numerous superior properties such as a short setting time, rapid development of mechanical strength, strong bonding strength to various substrates, and very good resistance to high temperatures. Hence, MPC was used to replace epoxy resins in the fabrication of fiber-reinforced polymer composites (FRP) with improved fire-resistance [3] or for the preparation of fire-retarding coatings applied on plywood [4,5].

Calcium magnesium phosphate cements (CMPC) can be obtained if magnesia is replaced with calcined dolomite. The conditions of the thermal treatment applied to natural dolomite have an important influence on the properties of the corresponding CMPCs. 

If the calcination of natural dolomite is performed in air at a relatively low temperature (1200 °C), the reactivity of the resulting oxides (CaO and MgO) is very high, thus when mixed with a phosphate (KH_2_PO_4_) solution, an important increase in system temperature and expansion of the paste is recorded [6]. The increase in the calcination temperature to 1400 °C determines a reduction of the CaO and MgO reactivity, thus it is possible to obtain CMPC cement with improved mechanical properties when mixed with a KH_2_PO_4_ solution at an adequate dosage [6]. Another alternative to reduce the reactivity of CaO and MgO oxides, while keeping the thermal temperature at 1200–1300 °C, is to calcinate a mixture of natural dolomite and silica [6,7] or dolomite with bauxite and gypsum [8]. The phosphate cement based on this type of calcined dolomite and phosphate salts (NH_4_H_2_PO_4_ or KH_2_PO_4_) exhibits a steady strength increase vs. time and adequate volume stability. 

Chong et al. [9] reported that the addition of limestone into the MPCs based on dead burnt magnesia and KH_2_PO_4_ determines a decrease in the mechanical strengths, irrespective of the curing medium (air or water). This is correlated with a significantly negative influence exerted by limestone addition on K-struvite crystal growth and its main characteristics. 

Baghriche et al. [10] prepared MPC cements using partially calcined dolomite and NH_4_H_2_PO_4_, with/without cement kiln dust addition (0–30.wt%). The calcination of dolomite was performed at 720 °C, thus the resultant material contained mainly MgO and CaCO_3_. The authors reported that the addition of cement kiln dust up to 20 wt.% reduced the apparent porosity of this type of MPC paste and improved the mechanical properties and bond strength of old concrete. 

The present study assesses the possibility of preparing fire-resistant coatings for structural steel, starting with KH_2_PO_4_ and dead burned magnesia or dolomite calcined at 750 °C. To the best of our knowledge, the preparation and characterization of CMPC fire-protective coatings based on partially calcined dolomite is reported for the first time in this study.

## 2. Materials and Methods

The materials used in this study were:

- Magnesite calcined at 1500 °C (M)—industrial product (Tremag, Tulcea, Romania) [6,11];
- Dolomite calcined at 750 °C for 3 h (D_750_); the natural dolomite (Rodbungrup, Bucharest, Romania), submitted to this thermal treatment, had a content of 47% CaCO_3_ and 37.5% MgCO_3_ [6];
- KH_2_PO_4_ (MKP)—chemical reagent (Sigma-Aldrich, Darmstadt, Germany);
- Sodium tetraborate decahydrate (Na_2_B_4_O_7_·10H_2_O)—chemical reagent (Sigma-Aldrich, Darmstadt, Germany);
- Tap water.

The compositions of phosphate cement are presented in Table 1.

MKP and sodium tetraborate decahydrate (for certain compositions) were mixed with water, followed by the addition of calcined magnesite (M) or calcined dolomite (D_750_). The resulting paste was poured in molds or used to cover metals plates, as will be further presented.

The X-ray diffraction (XRD) patterns were obtained in a 2θ range of 5–80, with a Shimadzu diffractometer XRD 6000 (Shimadzu, Kyoto, Japan), CuKα (λ = 1.5406 Ȧ) radiation; the scanning speed was 2 °/min. 

Differential thermal analysis (DTA) and thermogravimetry (TG) analysis were performed in air, with a heating rate of 10 °C/min using a differential thermal analyzer Shimadzu DTG-TA 51H (Shimadzu, Kyoto, Japan).

The microstructure of the studied materials was assessed using a Quanta Inspect F scanning electron microscope (1.2 nm resolution-Thermo Fisher—former FEI, Eindhoven, Nederland) with an energy-dispersive spectrometer (SEM-EDS). 

The compressive strengths were assessed on paste specimens (prisms with 60 mm × 15 mm × 15 mm—length × width × height); the pastes were hardened the first day in the mold, and afterwards demolded in the air at 20 ± 2 °C for different periods of time (1, 7, and 28 days). The compressive strength was determined using a testing machine (Matest, Treviolo, Italy); the loading was performed at a rate of 5 mm/minute, and a minimum of four values, assessed on specimens cured in similar conditions, were recorded. 

**Figure 1:**
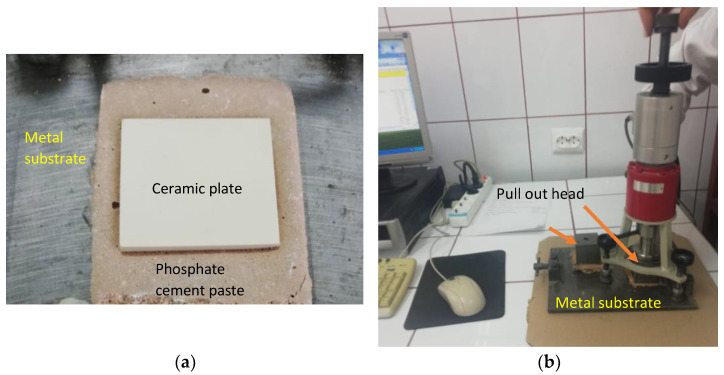
Pull-out test: (**a**) Preparation of test specimens; (**b**) test setup.

The tensile adhesion strength was determined on phosphate cement pastes applied to a metal substrate. A layer of cement paste (85 mm × 60 mm × 10 mm—length × width × height) was applied to a metal (steel) plate (previously sanded and degreased). On the top of this layer, a ceramic plate was placed (50 mm × 50 mm × 5 mm—length × width × height), and a supplementary mass (2000 g) was placed on the top, remained for 5 s, and then removed (Figure 1a). The specimens were cured in the air at 20 ± 2 °C for 1 day and 27 days. After this, a metallic pull-out head (cube—50 mm) was glued to the ceramic plate with an epoxy binder and cured for 14 h in air. The tensile adhesion strength was assessed with HZP 12 FORM +TEST machine (FORM + TEST Prüfsysteme, Riedlingen, Germany) for a direct-pull tensile force test by applying a force at a constant rate of 100 N/s (Figure 1b).

In order to compare this property of the studied phosphate cement with that of Portland cement (CEM I), a cement paste was prepared by mixing the cement with the amount of water corresponding to a water-to-cement ratio of 0.3 and applied to the metallic plate as previously presented.

The fire test was performed on steel plates (50 mm × 50 mm × 2.7 mm—length × width × height) to which a layer of phosphate cement paste was applied by tape casting (blading) and cured in the air at 20 ± 2 °C for 28 days. The plates were set vertically in a holder and the face covered with the phosphate cement layer was put in direct contact with a flame (propane burner) [12]. The temperature was assessed on the opposite side (back side) of the plate with a pyrometer. The accuracy of the pyrometer was ±1% from the recoded value +1 °C [12].

The mass loss was calculated with the following formula:

Δm= [(m_f_ − m_i_)/m_i_] × 100 (%) (1)

where:

m_i_ = the coating mass before the test (g).
m_f_ = the coating mass after the test (g).

## 3. Results

**Figure 2:**
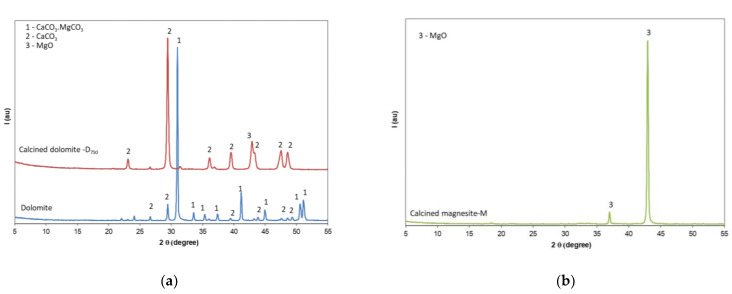
XRD patterns of (**a**) natural dolomite and dolomite calcined at 750 °C for 3 h (D_750_); (**b**) calcined magnesite.

The mineralogic compositions of calcined magnesite, natural dolomite, and dolomite thermally treated at 750 °C were assessed by X-ray diffraction (XRD). The XRD patterns presented in Figure 2 show the presence of MgO in calcined magnesite, CaCO_3_.MgCO_3_ and CaCO_3_ in natural dolomite, and MgO and CaCO_3_ in the dolomite calcined at 750 °C. As expected, the thermal treatment at this relatively low temperature determined the partial decomposition of dolomite (CaCO_3_.MgCO_3_) with the formation of MgO and CaCO_3_ [13,14,15].

**Figure 3:**
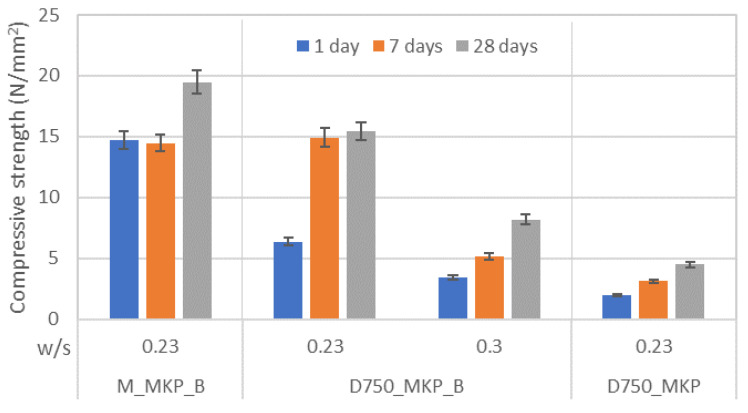
Compressive strengths vs. time for phosphate cement pastes based on calcined magnesite (M) or dolomite calcined at 750 °C for 3 h (D_750_), with water-to-solid ratios (w/s) of 0.23 and 0.3.

When calcined magnesite is mixed with a KH_2_PO_4_ solution, the main product assessed by XRD is K-struvite–KMgPO_4_·6H_2_O [11]. This compound is essential for the development of mechanical strength, as can be seen from the data presented in Figure 3. The phosphate cement based on calcined magnesite (M_MKP_B) has the highest values of compressive strengths for the studied time period. The compressive strengths of the phosphate cement based on calcined dolomite (D_750_) are smaller as compared to those of M_MKP_B, mainly due to the lower amount of MgO available for the formation of K-struvite. The use of a retarder (borax) improves the compressive strengths of phosphate cement based on D_750_, due to the increase in the setting time, and consequently, the maintenance of adequate workability of fresh paste for a longer time; also, the decrease in water dosage (from a water-to-solid ratio of 0.3 to a water-to-solid ratio of 0.23) further improves the compressive strength, most probably due to the reduction of porosity. 

**Figure 4:**
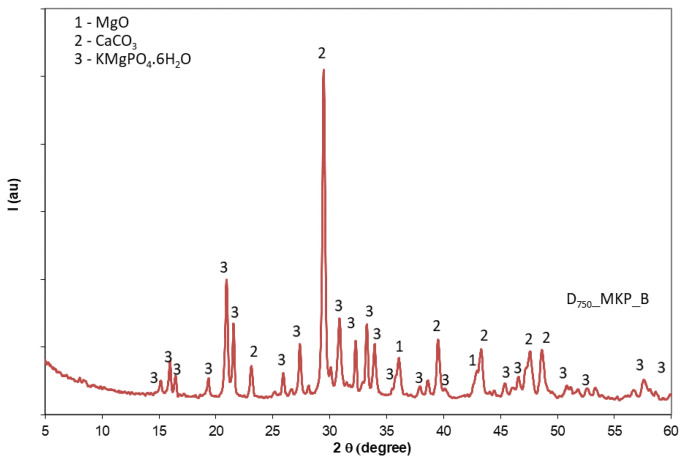
XRD patterns of phosphate cement based on calcined dolomite (D_750__MKP_B) with a water-to-solid ratio of 0.23.

The XRD patterns of the phosphate cement based on calcined dolomite (D_750__MKP_B) after 1 day of curing (Figure 4) show the presence of K-struvite along with CaCO_3_ and MgO.

**Figure 5:**
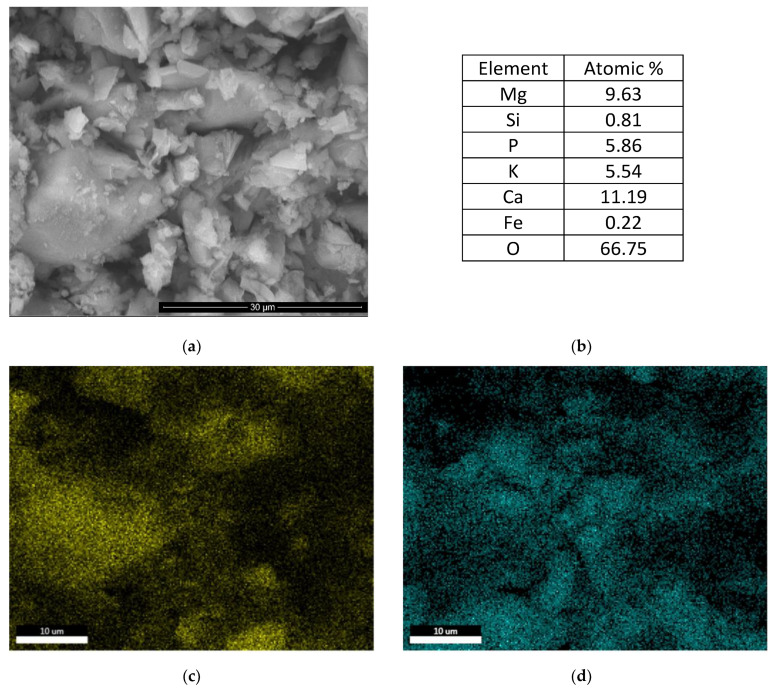

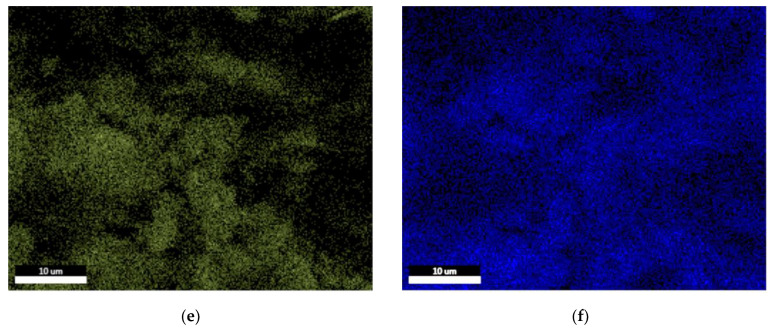
Electronic microscopy analyses of D_750__MKP_B (water-to-solid ratio = 0.23): (**a**) Back scattering electron image (×5000); (**b**) EDS analysis; elemental mapping: (**c**) Ca; (**d**) P; (**e**) Mg; (**f**) K.

The electronic microscopy analyses presented in Figure 5 confirm this composition for the phosphate cement based on calcined dolomite; the BSE image presented in Figure 5a shows the presence of big grains intermixed with small plate-like particles. The EDS analysis of this area shows the presence of calcium along with magnesium, potassium, and phosphorous. The atomic ratio of Mg:P:K = 9.63:5.86:5.54 suggests the presence of K-struvite along with CaCO_3_. The elemental maps presented in Figure 5c–f confirm the presence of Ca mainly in the big grains and Mg, K, and P in the smaller plate-like particles with a morphology specific to K-struvite i.e., triangular prisms [16].

The adhesion of the studied phosphate cement to metal/ceramic substrates was also assessed. The main results are presented in Table 2. As can be observed, the magnesium phosphate cement (M_MKP_B) has the highest adhesion to the metal substrate (failure occurred between the cement paste and ceramic tile glued to the pull-out head). The phosphate cement based on D_750_, with/without the borax addition, showed good adhesion to the metal substrate after 2 days of hardening. The adhesion strength to the metal substrate of D_750__MKP_B and D_750__MKP is higher as compared with the adhesion strength to the metal substrate of Portland cement (CEM I) paste (PC paste). The higher value of adhesion strength to the ceramic plate recorded after 2 days for the specimen with borax (D_750__MKP_B) as compared with the one without (D_750__MKP) could be due to initially better workability of the first paste. For longer curing periods (28 days), the adhesion to the metal substrate of the phosphate cement based on calcined dolomite decreases. Therefore, the fire test was performed on specimens cured for 28 days in similar conditions. 

**Figure 6:**
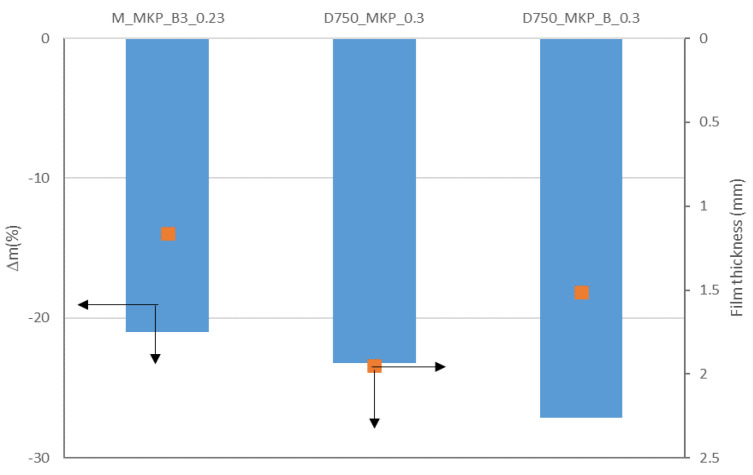
Film thickness (points) and mass loss—Δm (columns) after fire test for the studied coatings prepared with different water-to-solid ratios (0.3 and 0.23).

In order to evaluate the behavior of these coatings when put in direct contact with a flame, the back-side temperature of the metal plate covered with the studied cement pastes and the corresponding mass losses were recorded. As expected, the D_750__MKP_B coating prepared with a lower water-to-solid ratio (0.23) had a high thickness i.e., 2.36 mm (due to a high initial viscosity of the paste); therefore, we also prepared a coating with a higher water-to-solid ratio i.e., 0.3 (D_750__MKP_B_0.3). The mass losses (Figure 6) are mainly due to the water loss from K-struvite [16] as well as the decarbonization of calcite, as will be further presented.

**Figure 7:**
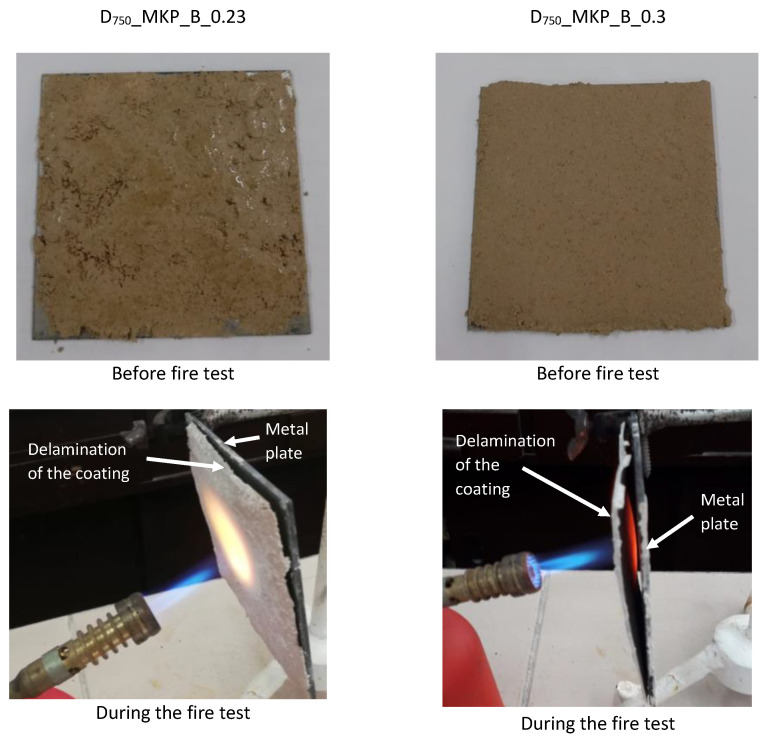
The visual aspect of metal plates with phosphate cement coatings (D_750__MKP_B, with water-to-solid ratios of 0.23 and 0.3) and their behavior during the direct flame test.

The coating based on calcined dolomite with the borax addition (D_750__MKP_B) shows important delamination from the metallic support during the fire test (Figure 7). For these specimens, the coating failed from the surface of the metallic plate after approximately 2 min of contact with the flame. A possible explanation for this behavior could be the formation of a lower amount of K-struvite (due to the delay determined by the borax addition) as well as the reaction of CaCO_3_ with KH_2_PO_4_ still present in the system with a negative influence on the mechanical properties [9]. 

**Figure 8:**
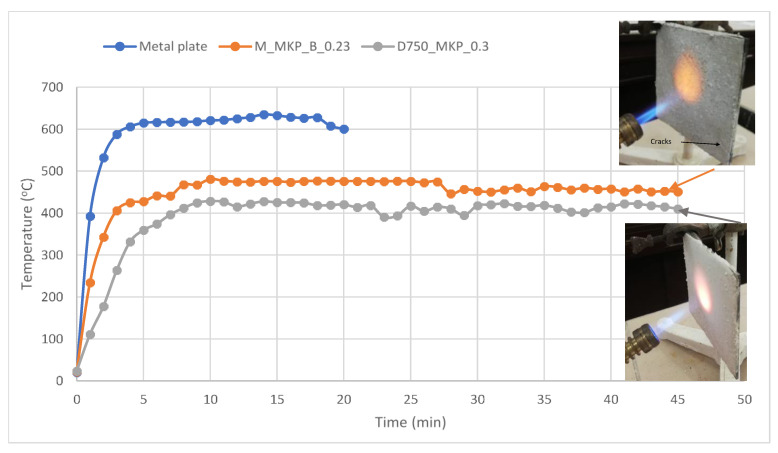
The visual aspect of metal plates with phosphate cement coatings and back-side temperature of metal plate without/with phosphate cement coatings, during the direct flame test.

**Figure 9:**
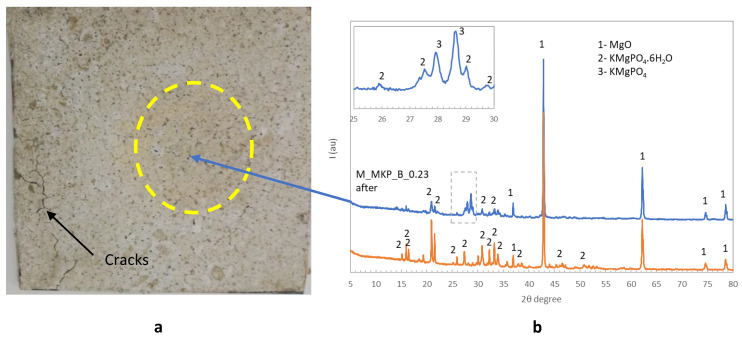
The visual aspect of M_MKP_B_0.23 coating after direct flame test (**a**) and the XRD patterns of the coating before and after the test (**b**).

A much better behavior was observed for the phosphate cement based on dolomite without the borax addition (D_750__MKP_0.3) when put in direct contact with the flame i.e., the coating adhered to the surface of the metal plate during the entire test period (45 min) and maintained a back-side temperature below 500 °C (the temperature considered critical for this type of structure [1,12])—see Figure 8. Similarly good behavior during the flame test was noticed for the coating based on calcined magnesite M_MKP_B_0.23 (Figure 8), although in one corner of the plate, one can notice the presence fine cracks that formed during the flame test (Figure 9a). Still, it is worth noting that the zone that was in direct contact with the flame (dotted circle in Figure 9a) presented no delamination and was crack-free.

The XRD patterns of the coating before the flame test show the presence of K-struvite together with MgO—Figure 9b. The XRD patterns of the material sampled after the direct flame test (from the dotted circle corresponding to the contact zone with the flame) shows certain compositional changes i.e., in the 2θ range 26–30 (see insert) new peaks specific to KMgPO_4_ [16] are also identified. This suggests that the main process that takes place during contact with the flame is the loss of water from K-struvite (KMgPO_4_·6H_2_O) with KMgPO_4_ formation; the identification in the XRD patterns of M_MKP_B, after the flame test, of the peaks specific to K-struvite (with low intensities) can be due to an impurification of the specimen with material from layers adjacent to the zone considered to be in direct contact with the flame.

**Figure 10:**
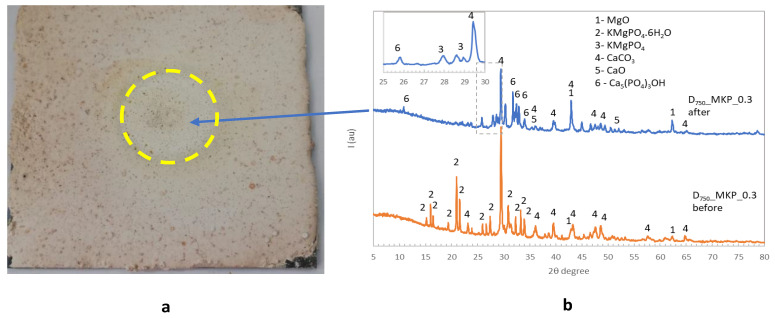
The visual aspect of D_750__MKP_0.3 coating after direct flame test (**a**) and the XRD patterns of the coating before and after the test (**b**).

The visual aspect of the coating based on calcined dolomite (D_750__MKP_0.3) after the flame test (Figure 10a) confirms its good behavior (no visible cracks, no exfoliation). The XRD patterns of the coating before the flame test show the presence of the main crystalline phases of CaCO_3_, KMgPO_4_·6H_2_O, and MgO; after the flame test, new peaks attributed to KMgPO_4_, Ca_5_(PO_4_)_3_(OH) (HAP), and CaO are also assessed in the XRD patterns (Figure 10b). Based on these results correlated with the literature [17,18,19,20] one can also consider the formation of HAP (or apatite phases [20]) with a low crystallinity degree in the reaction of the phosphate solution with calcium carbonate (at longer curing periods—up to 28 days) and its crystallization at the increase in temperature (during the flame test). 

**Figure 11:**
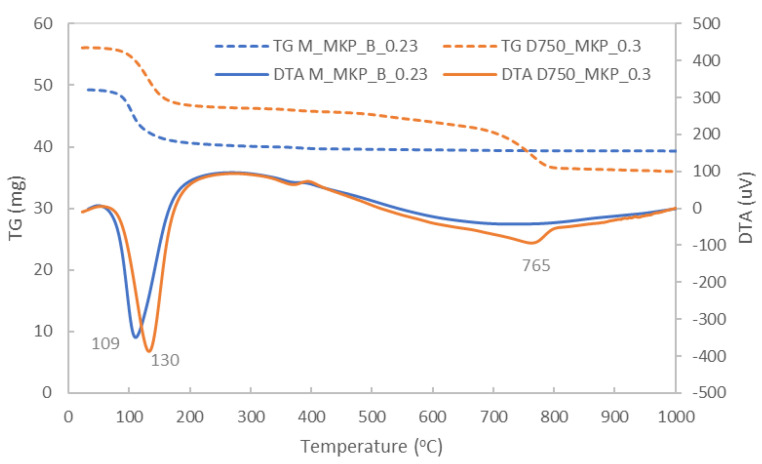
TG and DTA curves of M_MKP_B_0.23 and D_750__MKP_0.3.

DTA and TG analyses of M_MKP_B_0.23 and D_750__MKP_0.3 are presented in Figure 11.

On the DTA curve of magnesium phosphate cement (M_MKP_B_0.23), one can assess an important endo effect with the maximum at 109 °C, for which the weight loss assessed on the TG curve is 18.21%. According to the literature [16], this endo effect is determined by the water loss from KMgPO_4_·6H_2_O. On the DTA curve of the CMPC (D_750__MKP_0.3), one can assess the endo effect with the maximum at 130 °C with a corresponding weight loss of 17.3% (on TG) and a second endo effect with the maximum at 765°C (with a corresponding weight loss on TG curve of 12.2%), attributed to the decarbonation of CaCO_3_. The calculated heat for the effect(s) present on the DTA curve, between 30 and 1000 °C, was −2169 J/g for M_MKP_B_0.23 and −3105 J/g for D_750__MKP_0.3, which suggest a good ability to prevent the heat increase of the metal plate for both studied cements.

## 4. Conclusions

Based on the experimental results obtained in this study, the following conclusions can be formulated:

- The partial calcination of dolomite at a relatively low temperature (750 °C) permits the obtention of a mixture of MgO and CaCO_3_. The calcium magnesium phosphate cements (CMPC) resulting when partially calcined dolomite is mixed with a KH_2_PO_4_ solution contains the main crystalline compounds MgO, CaCO_3_, and K-struvite.
- The adhesion strength of magnesium phosphate cement (MPC) paste (based on dead burned magnesite) to a metal substrate is higher as compared to that of a ceramic substrate. The calcium phosphate cements (based on partially calcined dolomite) had better adhesive strength to the metal substrate as compared with the Portland cement paste after a short period of curing (2 days).
- The coatings based on MPC and CMPC, applied to a metal plate, were tested in direct contact with flame; the coatings of MPC and CMPC without the borax addition prevented the temperature increase of the metal substrate over 500 °C (considered critical for steel strength); moreover, during the entire period of the test (45 min), no exfoliation was noticed i.e., the coatings had good adhesion to the metal substrate.
- The results are promising but the study should be extended, also considering various additions to MPC and CMPC, aiming to improve the workability in fresh state as well as the adhesion to a metal substrate or/and fire resistance of these coatings.

## Figures and Tables

**Table 1:** Compositions of studied phosphate cement.

Sample	Calcined Magnesite (M) %wt.	Calcined Dolomite (D) %wt.	KH_2_PO_4_ (MKP) % wt.	Na_2_B_4_O_7_·10H_2_O * (B) %wt.	Water to Solid ** Ratio (wt.)
M_MKP_B	66.7	-	33.3	5	0.23
D_750__MKP_B	-	66.7	33.3	5	0.23 and 0.3
D_750__MKP	-	66.7	33.3	-	0.23 and 0.3

* calculated with reference to M (or D_750_) +MKP mixture; ** calculated considering also the water brought in the system by sodium tetraborate decahydrate.

**Table 2:** Assessment of adhesion strength to various substrates after 2 and 28 days of hardening; phosphate cement pastes were prepared with a water-to-solid ratio = 0.23 and PC paste was prepared with a water-to-solid ratio = 0.3.

Sample	2 Days			28 Days	
	Photo	Failure	Adhesion Strength (N/mm^2^)	Failure	Adhesion Strength (N/mm^2^)
M_MKP_B	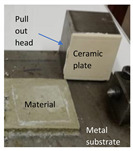	Interface ceramic plate–material	0	Interface ceramic plate–material	0
D_750__MKP_B	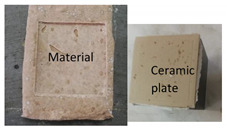	Interface ceramic plate–material	0.419	Interface metal– material	0
D_750__MKP	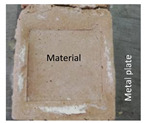	Interface ceramic plate–material	0.267	Interface metal–material	0
PC paste	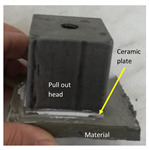	Interface metal–material	0	Interface metal–material	0

## Data Availability

Data sharing not applicable.
